# Healthcare professionals’ understanding of children’s rights: a systematic review of the empirical evidence-base

**DOI:** 10.1186/s13643-025-02756-9

**Published:** 2025-01-10

**Authors:** Sahar Mazied Alshammari, Mark A. Linden, Helen Kerr, Helen Noble

**Affiliations:** 1 Department of Maternal and Child Health, College of Nursing, University of Hail, Hail, 2440 Saudi Arabia; 2 Medical Biology Centre, School of Nursing and Midwifery, Queen’s University Belfast, 97 Lisburn Rd, Belfast, United Kingdom

**Keywords:** Children’s rights, The United Nations Convention on the Rights of the Child (UNCRC), Healthcare professional, Systematic review

## Abstract

**Background:**

The concept of children’s rights emerged during the 1980s and emphasised the role of children as active participants in matters which concern them.

**Aim:**

This review aims to identify and synthesise the empirical evidence base on healthcare professionals’ (HCPs) understanding of children’s rights.

**Methods:**

Five electronic databases (PubMed, CINAHL, Embase, PsycINFO, and the Web of Science) were systematically searched in May 2023. The Mixed Methods Appraisal Tool (MMAT) was used to quality appraise full-text papers included in the review. A descriptive narrative synthesis of the studies’ findings was performed.

**Results:**

A total of 15 relevant studies from 10 countries were identified and included in the review. The number of participants included ranged from 6 to 1048 for HCPs with a broad range of sampling methods. Based on the narrative synthesis of the included studies, three main themes were identified: (1) Barriers to implementing children’s rights in healthcare, (2) Factors that contribute to children’s rights implementation, and (3) Study instruments used to measure outcomes.

**Conclusions:**

HCPs require a better understanding of children’s rights to implement these rights into practice. Listening to children, building trusting relationships with children, and continuing professional development of HCPs could help to address barriers to understanding children’s rights. There is a pressing need for the development of a tool that is capable of tracking changes in the understanding of children’s rights in healthcare environments as efforts to increase awareness become more widely recognised.

## Introduction

Children’s rights have garnered significant attention and importance globally, particularly since the adoption of the United Nations Convention on the Rights of the Child (UNCRC) in 1989 [1]. This convention has been pivotal in acknowledging children as deserving of citizenship and has aimed to bolster the commitment of nations worldwide to uphold children’s rights [2]. Compliance with the UNCRC is overseen by the United Nations Committee on the Rights of the Child, which comprises independent international experts dedicated to safeguarding children’s rights [3]. The concept of children’s rights encompasses various facets, such as nurturance, self-determination, and participation rights, which are vital for safeguarding and fostering the development of children across physical, mental, emotional, social, moral, and economic domains [4, 5]. In recent years, there has been an increasing emphasis on aligning sustainable development goals with children’s rights agendas, as evidenced by the initiatives of the Welsh Government [6]. Moreover, Scotland has exhibited a strong dedication to integrating children’s rights both legally and in practice through programmes like ‘Getting it Right for Every Child’ [7]. This dedication mirrors a broader trend where children’s rights are progressively being acknowledged as indispensable elements of contemporary societies [5]. Furthermore, the significance of children’s rights education has been underscored as a crucial factor in advocating for and realising children’s rights [8]. In today’s healthcare institutions, treating children with dignity and respect, appreciating their competence, and supporting their choices are critical [9]. However, the participation of children in the healthcare context is rarely enabled in practice [10]. The HCPs play a vital role in upholding children’s rights in healthcare and are crucial in advocating for children’s rights, ensuring their active involvement in decision-making processes [11]. Incorporating children’s perspectives and preferences in healthcare settings is essential, as children often report not being able to participate in their healthcare decisions as much as they desire, despite having the legal right to express their views freely [12].

This discrepancy highlights the need to understand the extent to which HCPs respect the rights of hospitalised children, which is crucial for effectively implementing children’s rights and safeguarding their physical and psychological well-being in healthcare settings [8]. The HCPs play a crucial role in promoting the physical and mental well-being of children by addressing barriers that impede their access to optimal healthcare [13]. Despite recognising the value of talking with children, HCPs do not always seek or acknowledge children’s perspectives due to doubts about children’s understanding, time constraints, parental dominance in consultations, lack of training, institutional barriers, and fears of disrupting the consultation process [14]. The HCPs have the responsibility of advocating for children’s rights and providing guidance within the healthcare system. Therefore, this systematic review aims to identify and synthesise the existing empirical evidence base (HCPs) understanding of children’s rights.

## Materials and methods

### Search strategy

The Preferred Reporting Items for Systematic Reviews and Meta-Analyses (PRISMA) statement 2020 was employed to guide this review [15]. The search strategy was limited to studies published between 1989, the adoption of the UNCRC, and May 2023, the search date. Five electronic databases, PubMed, CINAHL, Embase, PsycINFO, and Web of Science, were searched. Key search terms were developed with the advice of a Queen’s University Belfast specialist subject librarian. The following search terms were used: (‘Child’s rights*’ OR ‘Child right*’ OR ‘Children’s right*’) AND (Healthcare professional* OR Healthcare provider* OR Health care worker* OR Healthcare practitioner* OR Health care personnel*). Terms were searched in the English language only. The combination of search terms and MeSH terms was used to maximise the total amount of literature retrieved. Boolean operators were also employed (OR, AND) to broaden keyword searches and then combine these. To achieve the aim of this systematic review, the research question was formulated using the SPIDER framework, What is the existing empirical evidence base on HCPs ' understanding of children’s rights?” which divides the question into the following components: Sample (S): HCPs, Phenomenon of Interest (PI): Understanding of children’s rights, Design (D): Studies with qualitative, quantitative, or mixed-methods designs that investigate understanding of children’s rights, Evaluation (E): Nature of understanding regarding children’s rights, Research type (R): Empirical studies which may include qualitative, quantitative, or mixed-methods research.

### Eligibility criteria

The following eligibility criteria were applied to the screening process to identify relevant papers which addressed our research question: Studies published from 1989 were included. English language studies and explored any children’s rights issues were included. Studies that involved HCPs as participants and focused on their understanding of children’s rights were considered. Only empirical research was included, while studies that included the views of non-HCPs, as well as other types of literature, such as systematic reviews, discussion or opinion papers, dissertations, and conference abstracts, were excluded.

### Selection of studies

**Fig. 1:**
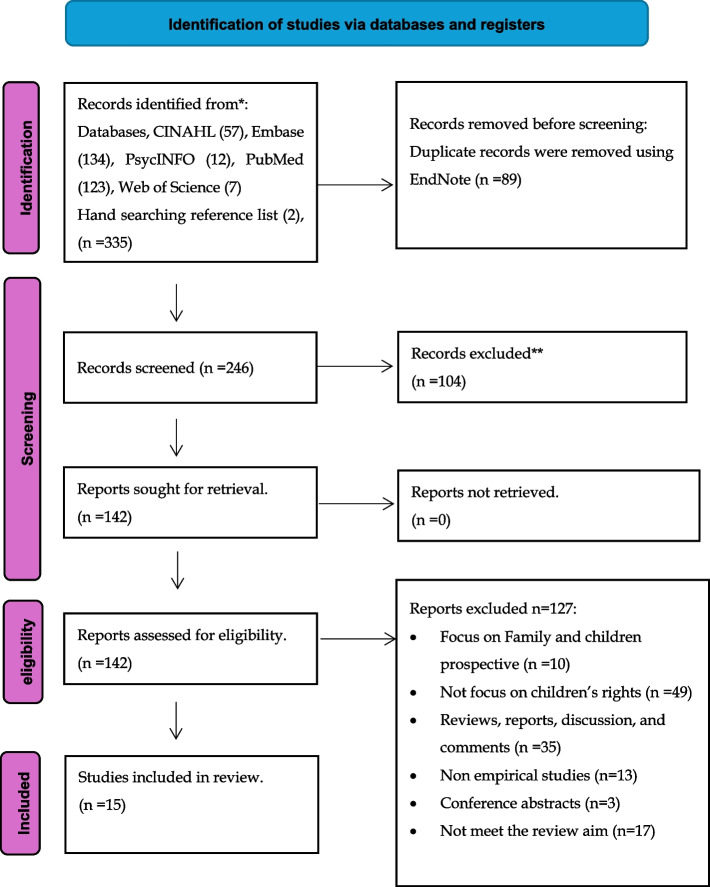
PRISMA flowchart

EndNote version 20 was used to manage references electronically and to remove duplicates [16]. A total of 301 studies were identified across five databases, and after removing 71 duplicates, a total of 230 studies were exported to Rayyan, an online systematic reviewing tool [17]. The title and abstracts of these studies were independently screened for eligibility by SA, ML, and HK using the stated inclusion and exclusion criteria. After the initial title and abstract screening, 126 full-text papers were read and discussed with SA, ML, and HK to determine suitability for inclusion in this review. At this stage, a total of 114 studies were excluded as they did not meet the eligibility criteria, resulting in 15 papers that met the aim of the systematic review. Two additional studies were added after hand searching [18, 19]. A PRISMA (2020) flowchart was used to display the process of study selection (Fig. 1). The final step was to extract the data from the included papers and perform a quality appraisal of the studies. Conflicts between authors were managed through initial discussions to clarify differing interpretations, and consensus was reached through collaborative deliberation. Only decisions agreed upon by all authors were included in the final analysis ensuring methodological rigour and consistency in the review process.

### Data extraction

To extract each study’s main characteristics, a pre-designed, standardised data extraction form was created and agreed upon by the research team. The data extracted from each study included identification data (the author’s name, publication year, and setting), methodological data (design of the study, aim, participants and measures), and the key findings (the children’s rights addressed, findings, and limitations). The data extraction was performed independently by SA, ML, and HK to ensure a rigorous process. Results were compared amongst the team for consistency. Any discrepancies were resolved by discussion and reappraisal until a consensus was reached.

### Quality assessment

The final included studies were scrutinised using the Mixed Methods Appraisal Tool (MMAT) to appraise the credibility, objectivity, and significance of the included studies [20]. The MMAT is used as a framework for the quality appraisal of identified studies of different methodological designs and, therefore, facilitates consistency of approach ([20]. The tool presents two screening questions for all types of studies and an explanation of the criteria for responding to ‘Yes’, ‘No’, or ‘Can’t tell’. Included studies were rated as ‘Strong’, ‘Moderate’, or ‘Weak’. SA, ML, and HK independently performed the quality assessment for all included studies.

### Data analysis, synthesis, and presentation

A descriptive narrative synthesis of the study findings was performed [21, 22]. The heterogeneity of study designs and outcomes precluded a meta-analysis, so we conducted a narrative synthesis [23]. We summarised the data extracted from the studies in text form and presented them in tabular format. The team then categorised the findings into three thematic groups based on their common characteristics, with the frequency of studies also presented. All authors were involved in the selection, review, and analysis of the papers. Themes were discussed among the team, and the final categorisation was agreed upon by consensus after reviewing all papers. One author drafted the narrative synthesis, which was subsequently reviewed and validated by all research team members.

## Results

### Characteristics of included studies

This review identified 15 studies that met the eligibility criteria. Table 1 provides a summary of the characteristics of the included studies, including research aim, design, sample, setting, children’s rights explored, and findings. Six studies employed a cross-sectional design [11, 19, 24–27], and eight used a qualitative design [18, 28–34]. Studies were completed in a variety of countries, including Ireland [18, 26, 33], Sweden [31, 34], South Africa [29], Italy [24], France [32], Switzerland [30], Canada [19], Turkey [11], and Nigeria [27], across all the UK ([28, 35], and Greece [25]. Sample sizes ranged from 6 to 1048 HCPs and utilised a broad range of sampling methods, including purposive, snowballing, and convenience.

**Table 1:** Characteristics of included studies

Author/year/country	Aim(s)	Sampling and setting	Design	Children’s rights explored	Significant Findings	Types of barriers	Facilitating factors	Suggestions for improvement
Adeleye et al. (2023) [27] Nigeria	To assess the knowledge, perception, and practice of child rights among doctors and nurses in Nigeria	Nonprobability sampling Sample: 821 HCPs Setting: (not mentioned)	Structured questionnaire (quantitative, cross-sectional)	Relevant laws on child labour*, child adoption*, criminal responsibility**, female circumcision**, and physical punishments**	−50% of HCPs are aware of child rights and adoption laws in Nigeria −70% of HCPs find no conflict between their beliefs and child rights legislation	Not mentioned in this study	Not mentioned in this study	–––-
Bester et al. (2018) [29] South Africa	To determine practices employed by South African HCPs to obtain consent for treatment from children	Purposive sampling Sample: *N* = 24 HCPs, (19 nurses, 5 medical practitioners) Setting: hospital and primary healthcare clinics	Semi-structured interviews (qualitative study)	Children’s consent**	-Inconsistent implementation of consent laws is due to limited knowledge, misinformation, and personal perceptions -HCPs explained that their current practices for assessing children's capacity to consent begin with building a trusting relationship and rapport, followed by providing information and conducting assessment practices	Practical barriers include limited time, staff shortages, and the need for multi-professional support and guidelines	Not mentioned in this study	-Increase staffing levels - Provide multi-professional training - Develop and disseminate clear guidelines - Promote team-based care and collaboration - Utilise supportive technology - Implement flexible scheduling for decision-making
Bisogni et al. (2015) [24] Italy	To understand the implementation and respect of rights in Italian paediatric hospitals	Sample: 536 nurses Setting: Paediatric hospitals and peadiatric units of general hospitals	Questionnaire (quantitative, cross-sectional)	Right to parental presence*, proper accommodation* play*, information on condition, assent/dissent, *privacy*, continuity of education*, pain management*	- The most implemented rights were related to parental presence (mean 4.47), play (mean 4.29) -The lower implemented rights were related to informed assent/dissent (mean 3.01), school education (mean 3.07) - The most neglected rights: pain treatment (16%), play and recreation (7.6%), treatment information (7.2%), privacy (5.2%)	Not mentioned in this study	Not mentioned in this study	––––
Clarke (2023) [35] United Kingdom	To explore children’s experiences of staying overnight in hospitals from the perspectives of children and nurses	Purposive sampling Sample: 8 children’s nurses Setting: Paediatric settings	Semi-structured interviews (qualitative study)	Caring for children hospitalised overnight**	-Three main themes were identified from interviews with children’s nurses: children’s nursing, job pressures, and safe and effective care	- Insufficient resources - Inadequate staffing levels - High patient care demands - Challenges in meeting children’s needs	Not mentioned in this study	- Increase resource allocation - Hire additional nursing staff - Implement workload management strategies - Provide specialised training to better meet children's needs - Enhance support systems for nurses (e.g. counseling, peer support) - Utilise time-saving technologies and tools
Coyne (2006) [28] England	To explore children’s, parents’, and nurses’ views on participation in care in the healthcare setting	Purposive sampling Sample: 11 children, 10 parents, and 12 nurses Setting: four paediatric wards in two hospitals	In-depth interviews, questionnaires, and observations (qualitative study)	Participation*	- Child’s on participation in care depends on age, attitude, well-being, illness length, mental status - Children’s participation requires parents’ approval - Workforce pressures challenge children's involvement in care decisions	Parents’ approval and Workforce pressures	Not mentioned in this study	- For parents’ approval issue: - Educate parents on benefits of child participation - Facilitate open discussions between HCPs, parents, and children - Provide decision-support tools for parents - For HCPs Workforce Pressures: - Increase staffing - Streamline workflows - Encourage team-based care
Coyne et al. (2014) [18] Ireland	To explore children’s participation in shared decision-making (SDM) from multiple perspectives	Purposive sampling Sample: 20 children, 22 parents, and 40 HCPs Setting: one haematology/oncology unit	Structured interview (qualitative study)	Participation in SDM*	- Children’s role in treatment decisions is limited	-Factors such as wellness, development, maturity, personality, emotional state, and disabilities can hinder children’s involvement - Constraints on children's participation in shared decision-making include: - Time limitations - Heavy workloads - Treatment urgency - Restricted choices	Not mentioned in this study	- Adapt communication to children’s developmental levels - Train staff on engaging children with diverse needs - Allocate more time for shared decision-making - Optimise workflows to reduce staff workload - Plan for children’s input even in urgent cases - Expand available choices for children in decision-making
Georgousopoulo et al. (2023) [25] Greece	To highlight the knowledge of employees in children’s hospitals regarding children’s rights	Purposive sampling Sample: 251 HCPs Setting: paediatric hospital	Questionnaire (quantitative, cross-sectional)	Access to health services without any discrimination*,necessary hospitalisation**, play and recreation*, education*, privacy*, pain management*	- The main findings indicate that less than 50% of HCPs are aware of the UNCRC and hospital-specific children's rights charters. Additionally, 53.9% of hospitalisations could have been avoided. A significant 88.3% of respondents support children's right to access information and participate in decision-making. Furthermore, 38.2% believe that gender and privacy are respected in multi-bed rooms. Regarding pain management protocols, 63.3% report partial or full usage, while 24.3% are unaware of them, and 12.4% state they are not followed	Not mentioned in this study	Not mentioned in this study	–––-
Lombart et al. (2020) [32] France	To explore HCPs perspectives on forceful physical restraint in paediatric care	Purposive sampling Sample: 30 female HCPs Setting: four paediatric hospitals	Ethnographic (qualitative study)	Autonomy*	HCPs experience significant fatigue and emotional strain when involved in restraining children	Fatigue and emotional strain	Not mentioned in this study	- Provide specialised training on managing difficult situations without restraint - Increase staffing to reduce individual burden - Implement de-escalation techniques and non-restraint alternatives - Offer emotional support and counseling for HCPs - Foster a team-based approach to share responsibility
Lutz (2019) [30] Switzerland	To analyse patients’ and professionals' conceptions of autonomy in childhood obesity management	Purposive sampling Sample: 8 HCPs, 29 children (aged 7–18 years) and 31 parents Setting: paediatric hospital	Semi-structured interview (qualitative study)	Autonomy*	- Lack of motivation linked to children's and parents’ attitudes -Professionals’ expectations of autonomy vary by age	Not mentioned in this study	Not mentioned in this study	––––
Migone et al. (2007) [26] Ireland	To assess perceptions of hospital adherence to EACH for Children in Hospital Charter	Purposive sampling Sample: 50 children, 111 parents, 61 nurses, and 41 doctors Setting: Dublin paediatric hospital	Questionnaire (Quantitative, cross-sectional)	The right to be protected from unnecessary medical treatment and investigations**The right of Parental accommodation within hospital settings**, right to be informed in a manner appropriate to their age and understanding*, rights of meeting children’s developmental and emotional needs during hospitalisation**	Many children undergo unnecessary treatments and can be treated at home. The hospital lacks adequate play and education facilities, and there is insufficient privacy on the wards. While over 90% of staff use appropriate language when discussing medical issues with parents and children, only 50% of nurses and 59% of doctors encourage children to ask questions. Additionally, 28% of nurses and 41% of doctors report having inadequate time to build relationships with parents and children	Not mentioned in this study	Not mentioned in this study	––––-
O’Connor et al. (2021) [33] Ireland	To describe perspectives on children’s participation in decision-making in therapy	Purposive sampling Sample: 7 children, 5 parents, and 6 occupational therapists Settings: public healthcare services	Semi-structured interview (qualitative study)	Participation in decision-making*	-Factors impacted childern participation in decision- making were as follows: attention/concentration, self-esteem, mental health, intellectual disabilities, speech/language difficulties - Empowering adults to empower children is crucial	Childern attention/concentration, self-esteem, mental health, intellectual disabilities, speech/language difficulties	Not mentioned in this study	- Use tailored communication aids - Provide mental health support - Boost self-esteem with positive reinforcement - Offer specialised support for intellectual disabilities
Quaye et al. (2021) [34] Sweden	To identify everyday situations illustrating the child’s best interests in medical care	Purposive sampling Sample: 32 children (aged 2–7 years), their parents, and HCPs Setting: one paediatric regional hospital and two paediatric units at a tertiary university hospital	Nonparticipant observation (qualitative study)	Best interest of the child*	- Facilitators to the expression of the child’s best interests: Provide introductory, preparatory, and sensory information, minimal parental interruption - Obstacles to the expression of the child’s best interests: communication barriers, parental interruptions, and lack of preparation time	- Communication barriers - Parental interruptions - Lack of preparation time	- Provide introductory, preparatory, and sensory information, minimal parental interruption	- Use clear, child-friendly communication tools - Educate parents on minimising interruptions - Allocate dedicated preparation time for HCPs - Implement structured communication strategies for HCPs and children
Ruiz-Casares et al. (2013) [19] Canada	To assess attitudes toward providing care to undocumented migrant children and pregnant women	Convenience sampling Sample: 1048 clinicians, administrators, and support staff Setting: 3 hospitals including 2 major paediatric hospitals and 2 primary care centres	Questionnaire (quantitative, cross-sectional)	Access to health services*	HCPs identified several barriers to providing care for undocumented migrant children and pregnant women. These include language barriers (79.2%), difficulty finding a doctor (72.1%), and fear of reporting to immigration authorities or deportation (59.4%)	-Difficulty finding a doctor -Fear of reporting to immigration authorities or deportation	Not mentioned in this study	- Create a referral network to simplify finding doctors - Educate HCPs on the rights and protections of undocumented children and pregnant women - Implement clear policies to reassure undocumented children about confidentiality - Offer legal support and guidance for migrant children and pregnant women in healthcare settings
Sahlberg et al. (2020) [31] Sweden	To investigate experiences of children and nurses in paediatric care of caring by the UNCRC	Convenience sampling Sample: 13 children (aged 4–7 years) and 11 nurses Setting: three paediatric primary healthcare units	Semi-structured interview (qualitative study)	Caring in accordance with UNCRC*	Nurses noted that investing time in preparing children for procedures facilitates future healthcare encounters. However, emergencies often limit opportunities for children to express themselves, leading to more parent-centred communication. Nurses try to compensate by allowing children to make small decisions related to procedures, such as choosing the colour of a bandage or expressing their fears	Not mentioned in this study	Not mentioned in this study	–––-
Yigitbas & Top (2020) [11] Turkey	To determine midwives’ and nurses’ attitudes toward their roles in implementing child rights in healthcare	Convenience sampling Sample: 122 midwives and nurses Setting: public paediatric hospital	Questionnaire (Quantitative, cross-sectional)	Best interest of the child*, Right to life and development Privacy*, Access to health information*, Parental responsibilities and state assistance*	Midwives and nurses often gauge a child’s understanding of treatment options through the child’s information (31.5%) or facial expressions (58.9%), while 36% believe children should not participate in health decisions. Midwives report higher rates of identifying and reporting suspected violence, abuse, and neglect	Not mentioned in this study	Not mentioned in this study	––––

*Right (s) aligned with the UNCRC

**Right (s) not aligned with the UNCRC

The study participants were all HCPs (nurses, midwives, doctors, social workers, dietitians, and occupational therapists). However, the numbers and types of each profession were not presented in some studies [30, 32, 34]. Four of the included studies [11, 24, 29, 32] emphasised exploring HCPs’ perspectives on children’s rights, while seven studies included the views of both parents and their children in addition to HCPs [18, 26, 28, 30, 31, 33, 34]. The included papers all reported a variety of children’s rights, such as participation in decision-making and consultation [18, 28, 31, 33], autonomy [30, 32], the best interests of the child [34], the right to receive care ([31], the right to access healthcare [19], the right to consent [29], and the right to receive care [35]. Moreover, several additional children’s rights were identified in the included studies, for example, the right to play, the right to continuity of education, the right to be free from pain, the right to be protected from unnecessary medical treatment, and the right to accommodation within hospital settings [24–26].

#### Quality assessment of the included studies

All the included studies were critically appraised using the MMAT Tool [20]. Nine studies were rated as being ‘Strong’ [18, 25, 28–30, 32–35], while six studies were rated as being ‘moderate’ due to inadequate reporting of details regarding nonresponse bias. The MMAT scores for each included study are presented in Table 2.

**Table 2:** Quality appraisal of included studies (MMAT)

Author and year	Study design	Q1	Q2	Q3	Q4	Q5	Q6	Q7	Comments
Adeleye et al. (2023) [27]	Quantitative	Y	Y	Y	Y	Y	CT	Y	Moderate
Bester et al. (2018) [29]	Qualitative	Y	Y	Y	Y	Y	Y	Y	Strong
Bisogni et al. (2015) [24]	Quantitative	Y	Y	Y	Y	Y	CT	Y	Moderate
Clarke, S (2023) [35]	Qualitative	Y	Y	Y	Y	Y	Y	Y	Strong
Coyne (2006) [28]	Qualitative	Y	Y	Y	Y	Y	N	CT	Strong
Coyne et al. (2014) [18]	Qualitative	Y	Y	Y	Y	Y	Y	Y	Strong
Georgousopoulo et al. (2023) [25]	Quantitative	Y	Y	Y	Y	Y	Y	Y	Strong
Lombart et al. (2020) [32]	Qualitative	Y	Y	Y	Y	Y	Y	Y	Strong
Lutz (2019) [30]	Qualitative	Y	Y	Y	Y	Y	Y	Y	Strong
Migone et al. (2007) [26]	Quantitative	Y	Y	Y	N	N	CT	Y	Moderate
O’Connor et al.(2021) [33]	Qualitative	Y	Y	Y	Y	Y	Y	Y	Strong
Quaye et al. (2021) [34]	Qualitative	Y	Y	Y	Y	Y	CT	Y	Strong
Ruiz-Casares et al. (2013) [19]	Quantitative	Y	Y	Y	Y	CT	CT	CT	Moderate
Sahlberg et al. (2020) [31]	Qualitative	Y	Y	Y	Y	Y	CT	Y	Moderate
Yigitbas and Top (2020) [11]	Quantitative	Y	Y	Y	Y	Y	CT	Y	Moderate

*Y* ‘yes’—This means the study clearly addresses the question. *N* ‘no’—This means the study did not clearly address the question. *CT* ‘Can’t tell’—This means the study reports unclear information related to the criterion.

#### Themes from the narrative synthesis

Based on the narrative analysis of the included studies, three themes were identified which addressed the study question around HCPs’ understanding of children’s rights: (1) Barriers to implementing children’s rights in healthcare, (2) Factors that contribute to the implementation of children’s rights and (3) Study instruments used to measure outcomes. Most of the studies’ findings related to more than one theme.

#### Theme 1: Barriers to implementing children’s rights in healthcare

This systematic review evaluated barriers affecting the implementation of children’s rights in healthcare settings, as reported in several studies [18, 19, 28, 29, 31, 33]. A recurring theme among these studies was the inconsistent application of children’s consent laws and the varied perceptions of HCPs regarding the appropriateness of obtaining consent from children. Bester et al. (2018) [29] conducted a qualitative study of South African HCPs’ practices in obtaining children’s consent and highlighted significant knowledge gaps and misconceptions about the Children’s Act (38 of 2005) in South Africa. While some HCPs were well-versed in the law, others displayed limited and sometimes incorrect knowledge, leading to inconsistent observance of consent laws. Additionally, there were differing perceptions about the necessity of obtaining consent from children, with some HCPs believing it was inappropriate to ask for consent. Bester et al. (2018) [29] concluded that HCPs were insufficiently prepared to assess children’s capacity to consent and recommended adequate training, multi-professional team support, and clear guidelines to assist in assessing children's mental capacity for consent. They also noted that labour shortages and high patient loads contributed to the inconsistent implementation of consent laws, as HCPs lacked sufficient time to engage with children adequately. Similarly, Sahlberg et al. (2020) [31] found that nursing staff believed providing child-friendly care required considerable time and resources, which they often lacked. The resulting stress and frustration prevented them from delivering optimal care. Clarke (2023) [35] supported these findings, noting that nurse participants experienced frustration and fatigue due to insufficient resources, staffing levels, and high patient care demands while trying to meet children’s needs. Coyne (2006) [28] concluded that the pressures of work led to substantial challenges for nurses in empowering children to express concerns about their care.

Barriers to children’s participation in healthcare decision-making were explored in several studies [18, 28, 33]. Coyne et al. (2014) [18] found that HCPs often did not engage children in Shared Decision Making (SDM) because the parameters of these decisions were typically well-defined, and optimal treatment decisions were considered crucial to a child’s survival. Another study by Coyne (2006) [28] indicated that children’s involvement in care decisions was heavily dependent on parental approval and the children’s cognitive maturity, specifically their ability to understand the rationale for their care. Two studies [18, 33] agreed that children with physical or intellectual disabilities were not involved in the decision-making process at the same level as children with typical cognitive development or no learning disabilities. O’Connor et al. (2021) [33] also highlighted that poor mental health, lack of concentration, and low self-esteem were additional factors impacting children’s participation in decision-making. Their study on occupational therapists’ perspectives found that these barriers significantly affected children’s engagement in the therapy process.

The UNCRC (1989) [1] mandates that the right to healthcare must be accessible to all children without discrimination. Ruiz-Casares et al. (2013) [19] conducted a large-scale study of 1048 HCPs in Canada to assess attitudes toward providing care to undocumented pregnant women and their children. They found that 79.2% of HCPs identified the language barrier as a major obstacle to patients and their carers receiving necessary information. Other significant barriers included difficulties in accessing primary healthcare (72.1%), understanding the healthcare system (66.8%), and fear of being reported to immigration authorities (95.4%). In summary, the studies reviewed highlight several common barriers to implementing children’s rights in healthcare settings. These include knowledge gaps and misconceptions about consent laws, resource constraints, high patient loads, and differing perceptions about children’s capacity to participate in decision-making. Additionally, specific challenges were identified for children with disabilities and those from marginalised communities, such as undocumented immigrants.

#### Theme 2: Factors that contribute to the implementation of children’s rights

Four studies explored the factors that facilitate children's rights in practice [11, 29, 33, 34]. Common among these studies was the recognition that empowering children through explicit arrangements and clear communication is crucial. For instance, O’Connor et al. (2021) [33] described how children were empowered through an explicit ‘power-sharing’ arrangement involving children, parents, and professionals. They found that listening to children in this context was essential in facilitating children’s participation in decision-making. Despite occupational therapists recognising the importance of children’s rights, there was a noted lack of knowledge regarding the significance of a child’s right to be heard and participate in decision-making. This practice was often guided by client-centred philosophies learned during professional training. Similarly, Quaye et al. (2021) [34] emphasised the importance of providing focused introductory information to children about their care to facilitate their best interests in the healthcare context. Examples included clarifying the roles of HCPs during hospitalisation, providing preparatory information before physical examinations, and informing children about the expected duration of their hospitalisation. Additionally, allowing children access to sensory information, such as touching and holding the blood pressure kit, was thought to be beneficial. In another study, Bester et al. (2018) [29] highlighted the necessity of creating a trusting relationship and sharing information with children as precursors to positive interactions and essential when assessing children’s capacity. In their qualitative study, they found that trust and communication were fundamental in obtaining consent from children younger than 12 years.

Three studies examined the potential benefits and drawbacks of children’s participation in decision-making in healthcare matters [18, 31, 33]. These studies underscored the significance of involving children in decision-making processes despite some reservations from HCPs. Coyne et al. (2014) [18] conducted a quantitative study emphasising that involving children in the decision-making process helps build trusting relationships and improve collaboration. They noted that while some HCPs believed it was not always useful to offer children treatment choices due to the children’s potential inability to refuse treatment, involving children still had significant benefits. O’Connor et al. (2021) [33] explored occupational therapists’ experiences with children’s decision-making and goal-setting in their qualitative, inductive study. They found that goal setting was often an informal procedure involving information exchange, dialogue, and shared decision-making. Occupational therapists asserted that negotiated decision-making, which included the child’s voice, had a significant positive influence on children’s goal achievement. Sahlberg et al. (2020) [31] highlighted that children and young people’s participation in decision-making is key to improving their well-being and development. They found that children are actively engaged in their care only when they are well-informed and feel safe. This study echoed the sentiment that children’s active participation leads to better health outcomes and a sense of autonomy.

Comparing these studies reveals several commonalities and differences. The studies [29, 33, 34] highlighted the importance of clear communication and information-sharing with children as a means to empower them and facilitate their rights. O’Connor et al. (2021) [33] and Quaye et al. (2021) [34] specifically pointed out the role of focused information in enabling children to understand and participate in their care processes, while Bester et al. (2018) [29] emphasised trust-building as a critical factor. In examining children’s participation in decision-making, Coyne et al. (2014) [18], Sahlberg et al. (2020) [31], and O’Connor et al. (2021) [33] all found that involving children in decisions had positive outcomes. Coyne et al. (2014) [18] and Sahlberg et al. (2020) [31] both noted that children’s involvement in decision-making improves trust and well-being. However, Sahlberg et al. (2014) [18] also reported that some HCPs doubted the utility of offering children treatment choices. O’Connor et al. (2021) [33] reinforced the idea that children’s voices significantly contribute to successful goal-setting and achievement.

While these studies varied in methodology and specific focus, the overarching theme was clear: empowering children through informed, respectful, and participatory practices significantly enhances their rights and well-being in healthcare settings. Despite some HCPs’ reservations, the consensus across these studies supports the importance of children’s active involvement in their care. Overall, this systematic review found that the facilitation of children’s rights in healthcare settings is heavily reliant on effective communication, trust-building, and the involvement of children in decision-making processes. The studies reviewed consistently highlighted the positive impact of these practices despite some identified challenges and gaps in knowledge among HCPs.

#### Theme 3: Study instruments used to measure outcomes of implementation

This systematic review evaluated the instruments used to assess children’s rights among HCPs across six studies, with a focus on the content and foundations of the tools. The analysis revealed that most instruments were built on internationally recognised frameworks and guidelines, such as the European Association for Children in Hospital (EACH) Charter and the United Nations Convention on the Rights of the Child (UNCRC). For instance, Georgousopoulou et al. (2023) [25] designed a structured questionnaire with 37 items, grounded in two main sources: the WHO ‘Self-evaluation Model and Tool on the Respect of Children’s Rights in Hospitals’ and the EACH Charter. These frameworks cover key children’s rights such as access to healthcare, privacy, parental presence, and protection from abuse. The tool was divided into three sections: demographics, knowledge of the UNCRC, and respect for specific children’s rights. It was adapted to the local context with minor modifications.

Additionally, the study conducted a test–retest procedure, reporting Cohen’s kappa values between 0.745 and 0.869, which suggests a strong level of reliability. However, despite demonstrating good test–retest reliability, the study’s unclear translation process for the questionnaire raises concerns about its content and cross-cultural validity, particularly in diverse settings. The studies varied in survey design and achieved response rates, impacting the generalisability and robustness of their findings. Similarly, Migone et al. (2007) [26] developed a questionnaire based on the EACH Charter, specifically aligning it with its ten articles, which include principles related to hospital admission, informed participation, and privacy for children in healthcare settings. The instrument’s content focused on the implementation of children’s rights, such as parental presence and child involvement in decision-making. Notably, the questionnaire, comprising sections for demographics and perceptions of hospital practices, was administered to children, parents, nurses, and doctors. It was piloted to enhance clarity; however, despite this effort, the study did not report further validation or other psychometric assessments, raising concerns about the tool's overall reliability and applicability in different contexts.

Bisogni et al. (2015) [24] used a 12-item questionnaire to assess the implementation of hospitalised children’s rights in Italian paediatric units, focusing on nurses’ perceptions. The instrument was built on the EACH Charter and the Associazione Ospedali Pediatric Italiani (AOPI) Charter, which outlines rights related to parental presence, access to information, play, privacy, and pain management. Participants rated the frequency of these rights being respected on a scale from 1 (never) to 5 (always). Although the instrument was pilot-tested and demonstrated good internal consistency with a Cronbach’s alpha of 0.81, the study’s lack of evaluation of other psychometric properties, such as construct validity or responsiveness, limits a comprehensive understanding of the instrument’s overall reliability. Yigitbas and Top (2020) [11] employed the Child Rights Education for Professionals (CRED-PRO) programme, which developed a comprehensive instrument with 38 items to measure HCPs’ attitudes toward child rights in healthcare settings. The tool focuses on professionals’ responsibilities, including ensuring informed consent, child participation in decision-making, and respecting children’s privacy. However, although the questionnaire was based on a reputable curriculum, it did not undergo psychometric properties in the study, leaving its reliability and validity unverified. This lack of evaluation raises concerns about the reliability and validity of the collected data.

Furthermore, in Adeleye et al. (2023) [27], a 54-item structured questionnaire was employed to assess HCPs’ knowledge, perception, and practice of child rights in Nigeria. The instrument was built on international and national frameworks, including the UNCRC, the African Charter on the Rights and Welfare of the Child (ACRWC), and Nigeria’s Child Rights Act (CRA). It covered areas such as criminal responsibility, child labour, female circumcision, adoption, and physical punishment. The questionnaire was validated through a pilot study, and its reliability was confirmed through statistical analysis, demonstrating strong performance in measuring HCPs’ adherence to child rights laws. However, the small sample size and lack of detailed validation processes raise doubts about the instrument's psychometric robustness, limiting its effectiveness for broader generalisation. Ruiz-Casares et al. (2013) [19] also utilised an 18-item online survey to assess HCPs’ attitudes toward healthcare access for undocumented children and pregnant women. Developed by a multidisciplinary team with input from an advisory committee, the tool focused on areas such as demographics, professional exposure to diverse populations, perceived barriers to healthcare access, and attitudes toward entitlement to healthcare services. While the questionnaire’s foundation on principles of human rights and child development offered a strong basis, it did not undergo further psychometric evaluation beyond a pilot test. The study provided limited details on the overall validation process, raising concerns about the thoroughness and rigour of the content validation. Without a clear validation process, it is difficult to assess the tool’s accuracy and relevance to the target population.

While many of these instruments were grounded in strong theoretical frameworks, their validation processes varied. Bisogni et al. (2015) [24] assessed internal consistency, reporting a Cronbach’s alpha of 0.81, while Georgousopoulou et al. (2023) [25] evaluated test–retest reliability, providing Cohen’s kappa values. However, the lack of comprehensive psychometric evaluations, including content and construct validity or responsiveness, limits a full understanding of the instruments’ overall reliability and applicability. Ultimately, the instruments reviewed in these studies were primarily built on well-established international and national frameworks, focusing on children’s rights in healthcare settings. However, most studies lacked comprehensive psychometric testing, indicating a need for more rigorous validation processes in future research.

## Discussion

This systematic literature review has explored the current empirical evidence base related to HCPs’ understanding of children’s rights. A total of 15 studies were included, demonstrating the extent of children’s rights knowledge, attitudes, and practices from HCP’s perspectives. This is the first systematic review that explores HCPs’ perspectives on children’s rights. In the current review, six studies utilised several quantitative measures for assessing HCPs’ perspectives. Despite attempts to measure HCPs’ knowledge of children's rights, none of the included studies used a psychometrically validated measure [11, 19, 26, 27]. Goldhagen et al. (2020) [36] emphasised the importance of the European Association for Children in Hospitals (EACH) Charter in advancing a child rights-based approach to child health and well-being, aligning with the UNCRC. The EACH guidelines describe the underpinning rights and standards of care that should be applied in healthcare settings. However, the assessment of the extent to which these standards have been successfully implemented cannot be measured without the development of a psychometrically validated tool.

Concerning scale validity and reliability, the recent scale developed by Georgousopoulos et al. (2023) [25] to evaluate employees’ knowledge of children’s rights in hospitals has demonstrated good face and content validity. It is considered acceptable in comparison to other non-psychometrically tested scales; however, no currently published study has sought to examine the factorial structure of this questionnaire. Indeed, it has yet to be tested for internal reliability. Hence, a notable gap in current research is the absence of a psychometrically tested questionnaire aligned with UNCRC, which could help to ensure an accurate assessment of HCPs’ understanding of children’s rights.

This review has identified many barriers to implementing children’s rights in healthcare contexts, including limited professional’ knowledge attitudes of HCPs and parents toward children, adult perceptions of a child’s vulnerability, and lack of resources [18, 28, 33]. As the implementation of children’s rights in hospitals affects outcomes and improves care, an improved understanding of these rights has become necessary. [37] The findings of this review identified a lack of knowledge as a barrier to implementing children’s rights (Bester et al., 2018). This is supported by [3], who confirmed that the lack of awareness of educational practitioners about some of the UNCRC’s articles is the cause of their inability to be implemented. Furthermore, in a review of knowledge of, and attitudes towards, children’s rights among HCPs, Pushpam et al. (2017) [38] demonstrated that adequate knowledge and application of children’s rights in healthcare was essential for high-quality services. This absence of awareness could be a result of a lack of training; therefore, the UNCRC (2008) has suggested providing systematic training for all professional groups working with children.

The implementation of children’s rights is also hindered by HCPs and parental attitudes towards children. In a Canadian study, Campbell and Katherine (2001) [39] found that one of the major barriers to implementing the UNCRC was a perception of children as family property. Similarly, the results from this review demonstrate that children’s involvement in the decision-making process is largely based on their parents’ permission [18, 28]. The HCPs were obligated to protect children by obtaining children’s consent and allowing children to communicate freely with their healthcare workers without their parent’s involvement [40]. Lundy (2007) [3] has stated that the fulfilment of children’s rights is dependent on how adults interact with children, particularly children’s rights to express their views on matters that affect them. Adult attitudes and beliefs regarding children were discovered to be the most important hurdles to the implementation of children’s rights in Wales [40]. Coyne et al. (2009) [41] emphasised that the actions of certain healthcare workers had effectively silenced hospitalised children, thereby denying them the opportunity to participate in research. Ensuring the engagement of these children in research is vital for the operationalisation of child-centred practice, as it facilitates the inclusion of their perspectives and experiences in the healthcare process. Adult perceptions of a child’s vulnerability are also a significant barrier to the implementation of children’s rights. In this review, HCPs believed that a child’s involvement in decisions depended upon the child’s age, ability, level of well-being, [18, 28, 33], and intellectual ability [33]. Tobin (2015) [42] argued that children's vulnerability does not offer a basis for rejecting children's rights, and these unique vulnerabilities justify the special rights guaranteed to children under the UNCRC. Ruiz-Casares et al. (2013) [19] examined HCPs’ attitudes toward providing care to undocumented migrant children and pregnant women in Canada. Ruiz-Casares and colleagues [19] found that language was cited by 79.2% of HCPs as the main obstacle preventing foreign-born children from accessing health services. In other words, migration brings linguistic diversity, which often results in language disparity between children and HCPs [43]. Although children are dependent on adults, HCPs should not ignore the need for their voices to be heard, and children’s rights should be respected regardless of their age and language.

In terms of guidance and regulations, obstacles to the implementation of children’s rights and the provision of their rights were highlighted in several studies [29, 34], particularly the necessity for specific legislation. Ju and Lee (2010) [44] highlighted the absence of a comprehensive system of laws in Korea to protect children and their rights. Similarly, the need for standard guidelines in implementing children’s rights was expressed by both [29, 34]. The lack of time and workforce pressures also constrained HCPs from involving children in decision-making [18, 28]. Similarly, Migone et al. (2007) [26] reported that 28% of nurses and doctors believed that they did not have adequate time for relationship-building with children. The provision of sufficient staffing could enhance children's satisfaction, address their rights, and gain their cooperation with the treatment plan.

A key finding of the review is that listening to children is crucial for the effective implementation of children’s rights. It has been stressed that listening to children is essential for improving service provision and ensuring that their feedback is considered in decision-making processes [45]. The UNCRC explicitly emphasises the right of children to share their opinions and be listened to, highlighting the significance of respecting children’s voices and perspectives [46]. The implementation of children’s rights requires the collection of high-quality data on children’s lives to identify disparities and variations in the realisation of their rights, emphasising the importance of actively listening to children’s experiences and perspectives [47]. In the context of healthcare, respecting children’s rights and ensuring their well-being necessitates HCPs listen to children and guarantee their rights to psychological and physical health [48]. Additionally, involving children in decision-making processes and listening to their voices is essential for understanding their experiences and addressing barriers or facilitators in accessing appropriate care [49]. Therefore, actively engaging with children in healthcare, respecting their voices, and considering their perspectives are fundamental aspects of upholding children’s rights and promoting their well-being and autonomy.

The HCP have a responsibility to facilitate children’s rights in their workplaces. George et al. [50] confirmed that HCPs should provide positive relationship experiences with children. Consequently, this leads to promoting children’s rights and helps children to develop personal ownership and identity in their beliefs, actions, and responsibilities [51]. When HCPs engage in respectful and supportive interactions with children, it can contribute to the development of a positive self-identity and a sense of responsibility for their well-being. This is in line with the UNCRC’s recognition of children as active agents in their own lives, capable of exercising their rights and taking on responsibilities as they develop. Therefore, the results from this review indicate how building trusting relationships with children and sharing information is an important component in implementing children’s rights [29]. Bell [52] underscores the importance of HCPs developing trust with children under their care to promote and protect children's rights and the role of corporate social responsibility in respecting and promoting children’s rights.

Continuous training on children’s rights is essential for HCPs to effectively uphold and enhance children’s rights in healthcare [53]. Training is crucial for professionals working with children to ensure that they have a comprehensive understanding of children’s rights and can effectively implement them in their respective fields [48, 54]. For instance, training programmes can increase the knowledge and awareness of children’s rights among HCPs in paediatric units, ultimately leading to better protection and advocacy for children in healthcare settings [48]. Moreover, training on children’s rights can have a positive impact on children themselves. Research has shown that teaching and supporting the rights of children through a rights education programme encouraged children to practice, protect, and promote the rights of others within their school, thereby fostering a culture of respect for rights from an early [55] Integrating children’s rights in early childhood education and care centres can contribute to creating a learning environment that respects and upholds children's rights, promoting their self-esteem and character development [56, 57]. It is crucial for professionals, including those working in healthcare, to stay updated on legislation and regulations that protect children’s rights to ensure that they are aligned with the latest developments [58, 59]. Ultimately, these findings highlight the critical need for comprehensive training for HCPs, improved resources, and clear guidelines to ensure that children’s rights are consistently upheld in healthcare settings.

### Strengths and limitations

This is the first systematic review that provides a comprehensive overview of HCPs’ understanding of children’s rights. This review employed a comprehensive search strategy, which was pre-designed and employed multiple databases. This ensured that the search was optimal, biases were minimised and guaranteed adequate and efficient coverage of the evidence base. In addition, a manual search of the reference lists of retrieved papers was also performed to ensure that no relevant studies were missed. Study selection and data extraction were completed independently and checked by two reviewers. This helped reduce errors in the data compiled for analysis. One limitation of our study is the inclusion of papers published only in English due to resource constraints. This may have led to the exclusion of relevant studies in other languages, limiting the generalisability of our findings. Additionally, we included studies from countries with diverse social and cultural contexts but did not fully explore how these differences may have influenced the results. Future research should account for these varying realities and consider including studies from a wider range of languages and cultural contexts to enhance generalisability. As the included papers were heterogeneous, it was impossible to compare results through meta-analysis; hence, a narrative analysis was undertaken.

### Implications for practice and future research

Further development of training programmes to enhance HCPs’ understanding of children’s rights is needed to adhere to the UNCRC’s articles. To enhance the understanding and awareness of children's rights, HCPs require targeted training interventions. Some of the included studies failed to fully capture the experiences of healthcare practitioners in this area. Further qualitative studies are needed to explore some of the areas identified in this review in greater depth. In addition, further evaluation of HCPs’ understanding of children's rights is required. For example, how they implement children’s rights in their contexts and how this may change over time. To achieve this and monitor progress, a psychometrically validated tool is required. This review identifies a gap in the literature concerning the understanding of children ‘s rights among HCPs. Previous studies have been unable to use valid and reliable instruments as none currently exist.

## Conclusions

In this systematic review, the primary quantitative and qualitative studies have been analysed to identify and synthesise the empirical evidence base on HCP’s understanding of children’s rights. Effective communication, trust-building, and child participation in decision-making were identified as facilitators of children’s rights in healthcare settings. In contrast, knowledge gaps and misconceptions about consent laws, resource constraints, high patient loads, and varied perceptions of children’s capacity for decision-making were identified as barriers. Additional challenges affect children with disabilities and those from marginalised communities, such as undocumented immigrants. To address these issues, the review underscores the necessity for comprehensive training for HCPs, improved resources and staffing, and the establishment of clear guidelines to ensure the protection and upholding of children’s rights in healthcare settings. Also, the studies included in this review failed to establish the validity and reliability of the instruments employed in evaluating HCPs understanding. Consequently, there is a pressing need for the development of a tool that is capable of tracking changes in understanding of children’s rights in healthcare environments as efforts to increase awareness are implemented. The implementation of the UNCRC is important, but without increased awareness and understanding, it will never be put into clinical practice. HCPs are major players in the healthcare system. Therefore, they must have an active role in the development, implementation, and understanding of children’s rights to ensure high-quality care for all children.

## Data Availability

Not applicable.
